# Development and validation of the perceived university employment support scale among undergraduate students

**DOI:** 10.3389/fpsyg.2025.1657774

**Published:** 2025-11-19

**Authors:** Jianmei Ye, Dawei Huang, Sujie Meng, Chunlan Long, Yukun Zhang, Ling Liu

**Affiliations:** 1 Key Laboratory of Adolescent Cyberpsychology and Behavior (CCNU), Ministry of Education, Wuhan, China; 2 School of Psychology, Central China Normal University, Wuhan, China; 3 Key Laboratory of Human Development and Mental Health of Hubei Province, Wuhan, China; 4 School of Humanities and Management, Guizhou University of Traditional Chinese Medicine, Guiyang, China

**Keywords:** university-based employment support, university graduates, social support theory, scale development, reliability and validity, career development

## Abstract

**Introduction:**

Grounded in social support theory, this study aims to develop and validate the Perceived University Employment Support Scale (PUESS) to systematically evaluate the role of higher education institutions in facilitating graduates’ successful transition into the labor market.

**Methods:**

Two studies were conducted to develop and validate the PUESS. Study 1 involved item generation, preliminary selection, and initial validation based on exploratory analyses. Study 2 further tested the construct validity, criterion-related validity, and measurement invariance of the finalized 20-item scale. Data were collected from diverse samples of university graduates to ensure representativeness.

**Results:**

The PUESS demonstrated strong internal consistency and robust construct validity across studies. The scale also showed comparable criterion-related validity to the University Student Social Support Scale and exhibited measurement invariance across gender, educational levels, and institutional types.

**Discussion:**

The findings provide empirical support for understanding how university-based employment support contributes to graduates’ career development. The PUESS offers a reliable and practical tool for assessing institutional employment support and provides actionable insights for education administrators and policymakers aiming to enhance employment services in higher education.

## 1 Introduction

The employment of young people, including university graduates, is closely tied to public welfare, economic development, and the nation’s future. The latest report titled “World Employment and Social Outlook: Trends to 2025” released by the International Labor Organization (ILO) (ILO, 2025) indicates that: In 2024, global employment expanded in line with a growing labor force, keeping the global unemployment rate steady at 5 percent, similar to that of 2023. At the same time, employment growth remained too weak to have a significant impact on persistent decent work deficits around the world. Young people, especially, continue to face much higher unemployment rates – around 12.6 percent – with few signs of improvements. Since 2020, the slowing of China’s economic growth, compounded by the pandemic and industry shifts, has significantly exacerbated the difficulties faced by university graduates in securing employment (Liu and Zang, 2024). Although China has effectively controlled the pandemic, the sustained impact has led to noticeable contraction and stagnation in sectors such as education and training, real estate, and the internet platform economy. This has resulted in a sharp reduction in hiring demand, especially from small and medium-sized enterprises, placing immense pressure on university graduates seeking employment (Li et al., 2021). Furthermore, data from China’s Ministry of Education indicates that the number of university graduates in 2025 is expected to reach a record high of 12.22 million (Ministry of Education, 2024).

In summary, university graduates face an increasingly challenging employment landscape, characterized by a rising number of graduates, slower economic growth, the lingering effects of the pandemic, and significant contractions in key employment sectors (Wang and Wang, 2024). These external and internal pressures have led to an exceptionally difficult job market for recent graduates. While the government continues to enhance employment services, imbalances between labor market supply and demand persist (Xiang et al., 2023). As institutions directly responsible for guiding graduates, universities play a pivotal role in providing employment support, which has become a critical component of student services (Barragán, 2017; Sun, 2020). However, current evaluations of the quality of university employment services remain inadequate, limiting progress in addressing graduate employment challenges. A thorough investigation into the quality of employment services at universities, alongside the establishment of an equitable evaluation system, is essential for improving service effectiveness. Such efforts would enable universities to refine and optimize employment strategies, allocate resources more efficiently, and match graduates with suitable positions, thereby facilitating a smoother transition into the workforce.

University-Based Employment Support plays a crucial role in the transition period for university graduates moving from academia to the workforce. It refers to the array of resources and services provided by universities to soon-to-be graduates, aiming to assist them in securing employment smoothly and achieving success in the early stages of their careers. In previous studies, most researchers have focused primarily on employment support for special student populations (e.g., individuals with autism, intellectual disabilities, and other impairments) (Chiang et al., 2013; McDonough and Revell, 2010; Wehman et al., 2014, 2015), with relatively little research on university-based employment support for graduates. Chinese scholars typically measure this support through single dimensions such as school support or teacher support within the broader context of social support for student employment (Wu, 2008). Moreover, some questionnaires exhibit poor psychometric properties (Wang, 2010; Zhang and Zhao, 2022) and complex structures. In brief, existing measurement tools lack integration, specificity, and professionalism, failing to comprehensively assess students’ perceived university-based employment support from aspects such as capacity building and psychological development.

Social support theory provides a robust theoretical framework for understanding institutional interventions in graduate employment. The theory posits that support from social networks significantly enhances individuals’ capacity to cope with stress and challenges through three primary mechanisms (Cohen and Wills, 1985; House et al., 1988; ILO, 2025) informational support involving career guidance and strategic advice, (Liu and Zang, 2024) instrumental support comprising concrete resources and practical assistance, and (Li et al., 2021) emotional support through psychological reinforcement.

Building upon this theoretical foundation, contemporary research has identified universities as pivotal institutional actors in delivering multidimensional employment support. Higher education institutions operationalize these support mechanisms through structured programs that mirror the tripartite framework of social support theory. For instance, career counseling services provide informational support by offering labor market insights, while internship placements exemplify instrumental support through direct workplace access (Ding and Jin, 2017; Rothwell et al., 2008).

Nevertheless, the unique role of universities as policy-making entities necessitates an extension of the classical social support framework. Unlike instrumental support–which emphasizes the provision of tangible resources such as internships, training, and facilities–policy support reflects institutionalized structural guarantees, regulatory frameworks, and procedural commitments grounded in institutional theory (Scott, 2014). Institutional theory suggests that formal structures, including rules, norms, and routines, shape behavior and legitimacy within organizations. Embedding policy support as a dimension thus underscores universities’ rule-setting and legitimacy-conferring functions in graduate support, beyond resource provision (Scott, 2014). Furthermore, perceived organizational support (POS) theory indicates that when institutions enact fair, consistent policies–such as transparent certification procedures or scheduling norms–it enhances recipients’ perceptions of support and trust in the institution (Eisenberger et al., 2001). These supportive policies can buffer stress and improve graduate outcomes.

Therefore, we argue that policy support is not merely an administrative derivative of instrumental support, but a structurally distinct and theoretically grounded dimension. It encompasses universities’ capacities to design and enforce institutional frameworks (e.g., employment subsidies, job application policies, graduation scheduling). Unlike instrumental support, which emphasizes tangible resources, policy support represents institutionalized structural commitments and procedural guarantees. Together with informational, instrumental, and emotional support, it constitutes a parallel and equally important dimension of perceived university employment support. This conceptual expansion strengthens the theoretical foundation of our study by reconciling a fundamental divergence from prior research: whereas most existing studies treat policy interventions as external contextual factors, our framework repositions policy support as a core dimension of employment support–consistent with institutional theory and POS–and central to a comprehensive understanding of university-based support.

This revised and theoretically substantiated framework directly informed the development of the Perceived University Employment Support Scale (PUESS). We aligned candidate items with the four dimensions–informational, instrumental, emotional, and policy support–ensuring conceptual clarity and empirical validity.

Integrating these dimensions, our study conceptualizes university-based employment support as a composite construct. Drawing from social support theory, we define perceived university-based employment support as graduates’ holistic evaluation of institutional assistance during school-to-work transition (Saks and Ashforth, 2002). This operationalization encompasses four interrelated components:

1. Informational support (career guidance systems)
2. Instrumental support (employment resource allocation)
3. Emotional support (psychological counseling services)
4. Policy support (institutional commitment frameworks)

In conclusion, based on the framework of social support theory, existing research seeks to develop a unified scale that meets psychometric standards–the PUESS–aimed at evaluating university graduates’ subjective perceptions of employment support from their alma mater. This questionnaire is expected to provide a reference for universities to improve employment support services and optimize employment guidance work. It will also enrich the empirical research tool system for assessing the quality of university employment services. Furthermore, the scale is anticipated to promote the development of related research efforts and enhance the overall level of employment services.

In order to establish the validity of the PUESS, we selected external measures grounded in existing theoretical and empirical research. For criterion-related validity, we adopted the Vocational Selection Anxiety Questionnaire for University Graduates (Zhang and Chen, 2006), as vocational selection anxiety is a well-established psychological outcome directly linked to employment stress during the school-to-work transition. Prior studies indicate that stronger institutional and social support are associated with lower levels of career-related anxiety, thereby making this scale a theoretically appropriate criterion measure (Wu and Zhao, 2024). For convergent validity, we employed the College Students’ Social Support Scale (Ye and Dai, 2008). Given that university employment support can be considered a domain-specific extension of broader social support, significant correlations with this general measure provide evidence that PUESS aligns with established constructs of perceived support, while still capturing unique employment-specific aspects. Together, these two instruments provide complementary evidence for the validity of the PUESS.

The present study aimed to develop and validate the PUESS within the framework of social support theory. Following standard scale development procedures, the research was conducted in three sequential phases.

**Phase 1: Item Development. A qualitative approach was employed to generate a comprehensive set of items.** Based on the theoretical dimensions of informational, instrumental, emotional, and policy support, an open-ended interview protocol was designed. Semi-structured interviews were conducted with 18 recent university graduates to explore their perceptions and experiences related to university-based employment support. The interview data were analyzed to construct an initial item pool. Subsequently, a panel of experts with doctoral backgrounds in psychology and education reviewed the items to evaluate their content relevance, clarity, and theoretical alignment, resulting in a refined preliminary version of the scale.

**Phase 2: Exploratory Factor Analysis (EFA).** The preliminary scale was administered to a large sample of university graduates. EFA was conducted to identify the underlying factor structure and to eliminate items with weak or cross loadings, leading to the development of a more concise and psychometrically sound version of the scale.

**Phase 3: Confirmatory Factor Analysis (CFA) and Validity Testing. CFA was conducted on a separate sample to validate the factor structure obtained in Phase 2.** To establish the robustness of the PUESS, we further tested its convergent validity, discriminant validity, criterion-related validity, and test–retest reliability. Measurement invariance was also examined across gender, educational levels, and institutional types to ensure applicability across diverse graduate populations.

This study was conducted in China, with participants recruited from various higher education institutions nationwide–including vocational colleges, general universities, and key national universities. The sample included job-seeking graduates from 12 major disciplines such as philosophy, economics, literature, and management. The findings offer strong empirical support for the reliability and validity of the PUESS and provide valuable implications for improving employment support practices in higher education settings.

## 2 Materials and methods

### 2.1 Participants and procedure

Sample A: Qualitative Analysis. It was selected using convenience sampling from 18 recent graduates across 16 universities in 8s of China, including 6 males and 12 females; 14 master’s students and 4 undergraduates; with an average age of 24.44 ± 1.29 years. Approximately 35-min semi-structured interviews were conducted with participants via online calls. This study received approval from the Ethics Committee of Central China Normal University. Informed consent was obtained from participants, with signed consent forms. A trained psychology graduate student conducted the interviews, clarifying that the data would be used solely for research purposes and that all personal information would remain confidential. Participants were compensated 15 RMB upon completion of the interviews.

Sample B: Used for item analysis and EFA. We employed convenience sampling to conduct an initial test with 183 graduates selected from multiple universities across China (including vocational colleges, general universities, and key universities), covering 12 major disciplines such as philosophy, economics, literature, and management. After excluding invalid questionnaires from non-fresh graduates and those not planning to seek employment, we obtained 160 valid questionnaires, resulting in an effective response rate of 87.43%. Among the participants, there were 60 males and 120 females; the educational levels were distributed as follows: 10 associate degree students, 70 undergraduates, 99 master’s students, and 1 doctoral student. The average age was 24.11 ± 2.16 years. Data collection was conducted online via Tencent Questionnaire. Before completing the questionnaire, participants were informed that the survey was voluntary and that they could withdraw at any time. An online informed consent form was provided on the survey’s homepage, informing participants that the survey was anonymous and that all data would be kept confidential.

Sample C: Used for CFA, reliability and validity testing, and measurement invariance analysis. The survey methods and scope were consistent with Sample B. A total of 436 graduates were conveniently sampled to participate in the formal test, and 400 valid questionnaires were ultimately obtained, resulting in an effective response rate of 91.74%. Among them, there were 168 males and 232 females; the educational levels were distributed as follows: 48 associate degree students, 190 undergraduates, 148 master’s students, and 14 doctoral students. The average age was 24.85 ± 2.34 years.

Sample D: Used for test-retest reliability analysis. Four weeks later, a second survey was conducted online with 110 graduates from Sample C, and 100 valid questionnaires were obtained, resulting in an effective response rate of 90.91%. Among them, there were 40 males and 60 females; the educational levels were distributed as follows: 8 associate degree students, 40 undergraduates, 48 master’s students, and 4 doctoral students. The average age was 24.58 ± 2.67 years.

### 2.2 Measurement

The Perceived University Employment Support Scale (PUESS). The primary instrument used in this study was the self-developed PUESS, designed to assess graduates’ perceptions of employment support provided by their universities. The scale consists of 20 items across four dimensions: Perceived Informational Support (5 items, e.g., “The university publishes job postings through multiple channels for us”), Perceived Instrumental Support (5 items, e.g., “The university’s industry-university cooperative enterprises meet my job search needs”), Perceived Emotional Support (5 items, e.g., “The university provides psychological counseling or consultation services related to employment”), and Perceived Policy Support (5 items, e.g., “The university is highly efficient in issuing various certification documents needed for job applications”). All items are rated on a 5-point Likert scale ranging from 1 (“Strongly Disagree”) to 5 (“Strongly Agree”), with higher total scores indicating a higher perceived level of university-based employment support. The development and validation process of the PUESS is the central focus of this paper.

The Vocational Selection Anxiety Questionnaire for University Graduates, developed by Zhang and Chen (2006), was employed to measure anxiety specifically related to career decision-making and job hunting. This scale has been validated for use with Chinese graduate populations. The Cronbach’s α coefficient of this scale is 70.867.

The College Students’ Social Support Scale, developed by Ye and Dai (2008), was used to measure general perceived social support. This scale assesses the support students receive from various sources (e.g., family, friends, others) and has demonstrated good reliability and validity in previous research. The Cronbach’s α coefficient of this scale is 0.906.

### 2.3 Data analysis

Data analyses were conducted using NVivo 12.0, IBM SPSS Statistics 28.0, and Mplus 8.0, corresponding to the three phases of the study.

In Phase 1 (Item Development), qualitative data from semi-structured interviews with graduates were analyzed using NVivo 12.0. A thematic analysis was conducted based on the social support theory framework, including informational, instrumental, emotional, and policy support. Interview transcripts were coded and categorized into themes, which guided the generation of the initial item pool. Redundant or ambiguous items were refined through iterative coding and expert review.

In Phase 2 (Exploratory Factor Analysis), item-level statistics (e.g., means, standard deviations, corrected item–total correlations) were computed using SPSS 28.0 to assess item quality. An exploratory factor analysis (EFA) with principal axis factoring and Promax rotation was then conducted to identify the underlying factor structure. The number of factors was determined by eigenvalues (>1), scree plot inspection, and parallel analysis. Items with low factor loadings (<0.40) or substantial cross-loadings were removed to improve the clarity and reliability of the scale.

In Phase 3 (Confirmatory Factor Analysis and Validity Testing), Mplus 8.0 was used to perform confirmatory factor analysis (CFA) to validate the factor structure identified in Phase 2. Model fit was evaluated using several indices: the chi-square statistic (χ^2^), comparative fit index (CFI), Tucker–Lewis index (TLI), root mean square error of approximation (RMSEA), and standardized root mean square residual (SRMR). Acceptable model fit was indicated by CFI and TLI ≥ 0.90, RMSEA ≤ 0.08, and SRMR ≤ 0.08.

To assess the construct validity of the scale, we examined convergent validity [using standardized factor loadings and average variance extracted (AVE)], discriminant validity (by comparing the square root of AVE with inter-factor correlations), and criterion-related validity through correlations with a validated measure of university student social support. Test–retest reliability was assessed over a 2-weeks interval using intraclass correlation coefficients (ICCs).

Finally, measurement invariance across gender, educational levels, and institutional types was tested using multi-group CFA in Mplus. Configural, metric, and scalar invariance were evaluated by comparing nested models, using changes in CFI (ΔCFI ≤ 0.01) and RMSEA (ΔRMSEA ≤ 0.015) to determine invariance across groups.

## 3 Results

### 3.1 Phase 1: items development

#### 3.1.1 Interview outline

To ensure the theoretical grounding and content relevance of the scale items, this study adopted social support theory as the conceptual foundation for item development. Drawing on the four core dimensions of social support–informational support, instrumental support, emotional support, and policy support–an open-ended interview outline was developed to explore graduates’ perceptions and experiences of employment support provided by their universities.

(1)    Informational Support
  - Are you familiar with all the channels through which the university releases job search information?
  - Do you believe the university provides sufficient employment information?
  - How well do these job postings align with your job search intentions?
(2)    Instrumental Support
  - Have the university’s job-search courses and activities been helpful to you?
  - Do the university’s partner companies meet your job search needs?
  - Has the career guidance counselor provided you with support?
  - Do you think the university lacks learning and communication platforms such as workshops on interview and test-taking skills, job information sharing sessions, or English speaking practice?
    ∘    If lacking, in which specific areas?
    ∘    If they exist, how have these platforms specifically helped you? (Please describe relevant scenarios).
(3)    Emotional Support
  - Does the university offer psychological counseling or consultation services related to employment?
  - Does the university proactively pay attention to students’ emotional states (e.g., anxiety, depression, stress)?
  - Has the university organized corresponding counseling or group guidance activities for students experiencing poor emotional states?
(4)    Policy Support
  - Does the university provide an overall graduation and job search plan?
  - During your job search, have the university’s related policies caused any disruptions for you?
  - How do you evaluate the efficiency with which the university issues various certification documents during the job search period?

#### 3.1.2 Construction of the initial scale

Each participant underwent an individual interview. Upon completion of the interviews, the audio recordings were transcribed into Chinese, with both the original recordings and initial transcripts preserved. Researchers meticulously reviewed the initial transcripts, repeatedly listening to the recordings to ensure accurate representation of the participants’ genuine expressions. During the text-cleaning process, words irrelevant or insignificant to the study were removed. To protect privacy, data from the 18 participants were coded according to gender (M for male, F for female) and numerical order, resulting in codes M1, M2, M3, M4, M5, M6, M7, M8, F1, F2, F3, F4, F5, F6, F7, F8, F9, and F10.

An expert team comprising a Ph.D. in Education, a Ph.D. in Psychology, and a Master’s degree holder in Psychology utilized Nvivo 12.0 to organize and analyze the verbatim transcripts totaling 100,000 words. Through saturation and validity testing, we obtained 142 first-level codes, 32 second-level codes, and 4 third-level codes–namely, Perceived Informational Support, Perceived Instrumental Support, Perceived Emotional Support, and Perceived Policy Support–which align with social support theory. Ultimately, based on the analysis of the interviews, we generated 32 items (8 items for each dimension).

To generate a more representative scale, the study invited a team of four psychometric experts–comprising one faculty member and three doctoral students–to evaluate and screen the item pool. The experts rated each item across three dimensions: whether it was accurate and clear, and whether it could lead to misunderstanding (0 = No, 1 = Yes) (Xu et al., 2024). Each expert independently assessed the items according to the criteria, and the team reached a consensus after discussion, ultimately retaining 24 items.

To evaluate the content validity, clarity, rationality, and readability of the items, the study invited an expert panel consisting of two psychology Ph.D. graduates and four psychology master’s graduates. The panel members first familiarized themselves with social support theory and relevant literature. Then, combining their theoretical knowledge and practical experience, they assessed the comprehensiveness, clarity, rationality, and readability of the items. For example, the item “The university’s employment services have played a critical role in helping me handle complex issues during the job application process” was deleted because it could be interpreted as the graduate’s personal feelings rather than support provided by the university. Ultimately, an initial questionnaire comprising 20 items across four dimensions was finalized. The questionnaire adopts a 5-point Likert scale, ranging from “Strongly Disagree” to “Strongly Agree,” scored from 1 to 5. A higher total score indicates that graduates perceive higher levels of university-based employment support.

### 3.2 Phase 2: EFA

The objective of this phase was to use SPSS 27.0 software to conduct item analysis and EFA on Sample B. This process aimed to optimize and refine the structure of the scale, ultimately yielding the finalized version of the PUESS.

#### 3.2.1 Item analysis

An item analysis was conducted on participants from Sample B using the PUESS. We employed independent samples *t*-tests for the top and bottom 27% of total scores (critical ratio method) and the item-total correlation method. Results from the critical ratio method indicated that all items had highly significant critical values (*P* < 0.001). The item-total correlation analysis showed that all items had item-total correlations above 0.45. All 20 items demonstrated good discriminative power and homogeneity.

#### 3.2.2 EFA

Firstly, we conducted EFA on the PUESS. The results showed that the Kaiser-Meyer-Olkin (KMO) value was 0.95, and Bartlett’s test of sphericity yielded a χ^2^ value of 3286.58 (*P* < 0.001). This indicates that the data were suitable for factor analysis.

Subsequently, we extracted principal components using the maximum likelihood method and performed varimax rotation to obtain the rotated component matrix. The criteria were eigenvalues greater than 1 and more than three items per factor. We examined whether each item’s factor loading exceeded 0.40 and communalities were not less than 0.30. Ultimately, all 20 items met these standards, forming the finalized scale. Four factors with eigenvalues greater than 1 were extracted, accounting for a cumulative variance contribution of 80.57%. Factor 1 was named Perceived Informational Support, Factor 2 as Perceived Instrumental Support, Factor 3 as Perceived Emotional Support, and Factor 4 as Perceived Policy Support. Each factor comprised 5 items. See Table 1 for details.

**TABLE 1:** Exploratory factor analysis (EFA) of the PUESS (*n* = 160).

Items	Factor				Communality
	1	2	3	4	
A3 The job postings provided by the university highly match my job search intentions.	0.86				0.68
A2 The university provides abundant job postings that meet my job search needs.	0.74				0.68
A5 The university promptly and accurately conveys relevant job search information to me.	0.72				0.93
A1 The university publishes job postings through multiple channels for us.	0.71				0.68
A4 The job postings provided by the university are highly timely.	0.66				0.64
B1 The university’s industry-university cooperative enterprises meet my job search needs.		0.86			0.89
B2 The university provides suitable job search courses based on my actual situation.		0.75			0.69
B3 The university offers learning and exchange platforms such as workshops on interview and test-taking skills, job information sharing sessions, and English speaking practice.		0.75			0.63
B5 University staff (career center staff, supervisors, counselors) provide me with practical job search advice (e.g., internships, job hunting).		0.73			0.72
B4 The university provides us with venues for online interviews.		0.64			0.73
C5 During my job search process, I feel warmth and support from the university.			0.80		0.69
C4 The university conducts lectures or forums on employment mental health.			0.73		0.66
C2 The university proactively understands everyone’s emotional state regarding employment.			0.72		0.67
C1 The university provides psychological counseling or consultation services related to employment.			0.71		0.70
C3 Communicating with university staff (psychological counselors, supervisors, counselors) helps me alleviate job search anxiety.			0.69		0.92
D1 The university is highly efficient in issuing various certification documents needed for job applications.				0.93	0.50
D3 The university plans graduation-related matters in advance, avoiding conflicts with my job search plans.				0.66	0.54
D2 The university’s relevant policies have not negatively affected my job search.				0.62	0.99
D4 The university’s employment policies greatly assist me in finding a job.				0.61	0.54
D5 The university has formulated corresponding employment policies to help us find jobs better.				0.60	0.53
Eigenvalues	12.09	1.70	1.28	1.04	
Variance contribution (%)	60.45	8.51	6.40	5.20	

It is noteworthy is that, in operationalizing policy support, we differentiate between universities’ structural commitments (e.g., policy design and institutional guarantees) and administrative execution (e.g., efficiency in issuing certificates). The items (D1–D5) were thus constructed to capture both the procedural dimension (D1–D3) and the institutional commitment dimension (D4–D5), reflecting the dual nature of policy support as distinct from instrumental assistance.

### 3.3 Phase 3: CFA and validity tests

#### 3.3.1 CFA

Based on the results of EFA, a second-order single-factor model was constructed–that is, it was assumed that the four first-order factors form one second-order factor. CFA was conducted on this model using Sample C. The results showed that the fit indices were χ^2^/*df* = 2.63, CFI = 0.96, TLI = 0.95, RMSEA = 0.05, and SRMR = 0.06. All model fit indices met psychometric requirements, indicating that the theoretical model of the PUESS fits well with the sample data and that the overall model fit is good. The structure of the PUESS

aligns with theoretical expectations and demonstrates good construct validity.

#### 3.3.2 Correlations between total scores and factor scores

A Pearson correlation analysis was conducted on the data collected from Sample C. The results showed that the total score of the PUESS was significantly correlated with each factor score, with correlation coefficients ranging from 0.76 to 0.81. The correlation coefficients among the factors ranged from 0.44 to 0.55. See Table 2 for details.

**TABLE 2:** Correlations between total score and factors, and among factors (*n* = 400).

Factor	1	2	3	4
1. Perceived Informational Support	–			
2. Perceived Instrumental Support	0.52^***^	–		
3. Perceived Emotional Support	0.47^***^	0.44^***^	–	
4. Perceived Policy Support	0.55^***^	0.53^***^	0.45^***^	–
Total scale	0.81^***^	0.80^***^	0.76^***^	0.78^***^

^***^P < 0.001; the same applies below.

#### 3.3.3 Reliability analysis

##### 3.3.3.1 Internal consistency coefficients

The examination of Sample C revealed that the Cronbach’s alpha coefficients of the PUESS were as follows: total scale = 0.94, Perceived Informational Support = 0.92, Perceived Instrumental Support = 0.93, Perceived Emotional Support = 0.93, and Perceived Policy Support = 0.88. These results indicate that the scale has excellent internal consistency.

##### 3.3.3.2 Test-retest reliability

After matching Samples C and D, we conducted a test-retest reliability analysis. The test-retest reliability coefficients for the total scale and each factor were: total scale = 0.83, Perceived Informational Support = 0.76, Perceived Instrumental Support = 0.80, Perceived Emotional Support = 0.73, and Perceived Policy Support = 0.66. These findings suggest that the scale possesses good stability.

#### 3.3.4 Validity analysis

##### 3.3.4.1 Criterion-related validity

We employed the Vocational Selection Anxiety Questionnaire for University Graduates (Zhang and Chen, 2006), as vocational selection anxiety is a well-established psychological outcome directly linked to employment stress during the school-to-work transition. The PUESS demonstrated a strong and significant negative correlation with vocational selection anxiety (*r* = −0.742, *p* < 0.01), indicating that higher levels of perceived university employment support are associated with lower levels of job-search-related anxiety. This result provides robust evidence for the criterion-related validity of the PUESS.

##### 3.3.4.2 Convergent validity

As shown in Table 1 above, all factor loadings of the PUESS exceed 0.5. According to Table 3, the Average Variance Extracted (AVE) values are all greater than 0.5, and the Composite Reliability (CR) values all exceed 0.7. This demonstrates that the PUESS has high convergent validity (Bagozzi and Yi, 1988; Fornell and Larcker, 1981). Furthermore, the College Students’ Social Support Scale (Ye and Dai, 2008) was employed. Results indicated a significant positive correlation between perceptions of university-based employment support and general social support (*r* = 0.325, *p* < 0.001), demonstrating that the PUESS is theoretically aligned with established measures of perceived support while retaining its unique employment-specific focus. This also demonstrates that the convergent validity of PUESS is quite good.

**TABLE 3:** Convergent validity and discriminant validity analysis (*n* = 400).

Factor	AVE	CR	1	2	3	4
1. Perceived Informational Support	0.72	0.93	(0.85)			
2. Perceived Instrumental Support	0.72	0.93	0.57^***^	(0.85)		
3. Perceived Emotional Support	0.72	0.93	0.51^***^	0.48^***^	(0.85)	
4. Perceived Policy Support	0.61	0.90	0.63^***^	0.60^***^	0.51^***^	(0.78)

Diagonal elements in parentheses represent the square roots of the AVE values. Off-diagonal elements represent the Pearson correlation coefficients between factors.

^***^p < 0.001.

##### 3.3.4.3 Discriminant validity

By comparing the square roots of the AVE values of each factor with the correlation coefficients between factors, we found that the minimum square root of AVE among the four factors is 0.78, which is greater than the maximum inter-factor correlation of 0.63. This implies that the PUESS has good discriminant validity (Fornell and Larcker, 1981; Wang et al., 2022).

#### 3.3.5 Single-group CFA (baseline model)

We conducted single-group confirmatory factor analyses (CFA) on the combined sample (Samples B and C), as well as across different genders (male, female), educational levels (undergraduate and below, master’s and above), and types of institutions (general universities, Double First-Class Universities), establishing single-group baseline models. As shown in Table 4, the four-factor model demonstrated excellent fit indices across the total sample, males, females, undergraduates and below, master’s students and above, as well as general universities and “Double First-Class” universities. The Comparative Fit Index (CFI) and Tucker-Lewis Index (TLI) ranged from 0.967 to 0.979, the Root Mean Square Error of Approximation (RMSEA) ranged from 0.046 to 0.053, and the Standardized Root Mean Square Residual (SRMR) ranged from 0.040 to 0.046. These values meet the criteria for excellent models as proposed by Cheung and Rensvold (1999). This indicates that the structural validity of the PUESS has been preliminarily confirmed under different contexts (Ye et al., 2025).

**TABLE 4:** Fit indices of the baseline model (*n* = 560).

Parameter	*n*	χ^2^	*df*	CFI	TLI	BIC	RMSEA (90% CI)	SRMR
Total sample	560	397.859	164	0.975	0.971	23108.526	0.050 [0.044, 0.057]	0.040
Male	238	272.423	164	0.972	0.967	13119.364	0.053 [0.041, 0.064]	0.046
Female	322	304.086	164	0.974	0.970	13273.414	0.052 [0.042, 0.060]	0.043
Undergraduate and below	353	296.930	164	0.976	0.973	14861.981	0.048 [0.039, 0.057]	0.043
Master’s and above	207	258.106	164	0.974	0.970	8559.072	0.053 [0.040, 0.065]	0.046
General universities	367	328.245	164	0.973	0.969	15245.864	0.052 [0.044, 0.060]	0.044
Double first-class universities	193	231.853	164	0.979	0.975	8139.008	0.046 [0.032, 0.060]	0.045

#### 3.3.6 Measurement invariance testing

##### 3.3.6.1 Measurement invariance based on gender

Measurement invariance models of the PUESS were established separately for male and female samples. As shown in Table 5, the Comparative Fit Index (CFI) and Tucker-Lewis Index (TLI) ranged from 0.969 to 0.976, the Root Mean Square Error of Approximation (RMSEA) ranged from 0.046 to 0.052, and the Standardized Root Mean Square Residual (SRMR) ranged from 0.044 to 0.046. All these indices met the criteria for good model fit. Further model comparisons revealed that ΔCFI, ΔTLI, and ΔRMSEA were all below the required thresholds of 0.01, 0.01, and 0.015 (Savickas and Porfeli, 2012). This indicates that the measurement structure of the PUESS items demonstrates strict invariance between male and female samples, and that gender factors do not have a significant impact on the application of the scale. These results support the good structural equivalence of the PUESS across different gender samples.

**TABLE 5:** Fit indices for each model based on gender variable (*n* = 560).

Model	χ^2^	*df*	CFI	TLI	BIC	RMSEA (90% CI)	SRMR
Configural invariance	576.509	328	0.973	0.969	23492.124	0.052 [0.045, 0.059]	0.044
Metric invariance	583.924	344	0.974	0.971	23398.291	0.050 [0.043, 0.057]	0.046
Scalar invariance	593.094	360	0.975	0.974	23306.214	0.048 [0.041, 0.055]	0.046
Strict invariance	603.134	380	0.976	0.976	23189.695	0.046 [0.039, 0.053]	0.046

##### 3.3.6.2 Measurement invariance based on educational levels

Measurement invariance models of the PUESS were established separately for different educational level samples (undergraduate and below vs. graduate and above). As shown in Table 6, the CFI and TLI for the four models ranged from 0.972 to 0.977, the RMSEA ranged from 0.045 to 0.050, and the SRMR ranged from 0.044 to 0.048. All indices met the criteria for good model fit. Further model comparisons revealed that ΔCFI, ΔTLI, and ΔRMSEA did not exceed the relevant thresholds of 0.01, 0.01, and 0.015, respectively (Savickas and Porfeli, 2012). This indicates that the measurement structure of the PUESS is invariant across different educational level samples, and that educational factors do not significantly impact the application of the scale. These results support the structural equivalence of the PUESS across samples with educational levels below undergraduate and those with graduate and above degrees.

**TABLE 6:** Fit indices for each model based on educational level variable (*n* = 560).

Model	χ^2^	*df*	CFI	TLI	BIC	RMSEA (90% CI)	SRMR
Configural invariance	555.036	328	0.975	0.972	23469.374	0.050 [0.043, 0.057]	0.044
Metric invariance	568.776	344	0.976	0.973	23381.868	0.048 [0.041, 0.055]	0.048
Scalar invariance	584.323	360	0.976	0.974	23296.167	0.047 [0.040, 0.054]	0.048
Strict invariance	593.441	380	0.977	0.977	23178.727	0.045 [0.038, 0.052]	0.048

##### 3.3.6.3 Measurement invariance testing based on school level

Measurement invariance models of the PUESS were established separately for samples from General Universities and Double First-Class Universities. As shown in Table 7, the Comparative Fit Index (CFI) and Tucker-Lewis Index (TLI) for the four models ranged from 0.971 to 0.977, the Root Mean Square Error of Approximation (RMSEA) ranged from 0.045 to 0.050, and the Standardized Root Mean Square Residual (SRMR) ranged from 0.044 to 0.045. All indices met the criteria for good model fit. Further model comparisons revealed that ΔCFI, ΔTLI, and ΔRMSEA did not exceed the respective threshold values. This indicates that the measurement structure of the PUESS is invariant across different school level samples (General Universities vs. Double First-Class Universities), and that school level factors do not significantly impact the application of the scale. Overall, these results support the good structural equivalence of the PUESS across samples from different school levels.

**TABLE 7:** Fit indices for each model based on school level variable (*n* = 560).

Model	χ^2^	*df*	CFI	TLI	BIC	RMSEA (90% CI)	SRMR
Configural invariance	560.098	328	0.975	0.971	23479.705	0.050 [0.043, 0.057]	0.044
Metric invariance	569.176	344	0.976	0.973	23387.535	0.048 [0.041, 0.055]	0.045
Scalar invariance	582.508	360	0.976	0.976	23299.621	0.047 [0.040, 0.054]	0.045
Strict invariance	594.87	380	0.977	0.977	23185.424	0.045 [0.038, 0.052]	0.045

## 4 Discussion

University-based employment support encompasses a comprehensive array of resources and initiatives aimed at facilitating graduates’ seamless transition into the labor market. It provides guidance, resources, and opportunities that assist students in navigating the challenges inherent in the job search process. These support systems are pivotal in enhancing students’ employability and fostering their adaptability within the competitive employment landscape. This study developed and validated the PUESS to systematically evaluate the impact of university-based employment support mechanisms on graduates’ employment outcomes.

This study systematically evaluated the reliability, validity, and measurement invariance of the PUESS, demonstrating that the scale possesses high psychometric quality. First, both qualitative research and EFA identified four dimensions within the scale: Perceived Informational Support, Perceived Instrumental Support, Perceived Emotional Support, and Perceived Policy Support. This structure aligns with social support theory and the theoretical model of university-based employment support (Cohen and Wills, 1985; House et al., 1988), indicating a solid theoretical foundation and good construct validity. Specifically, the Perceived Informational Support dimension reflects the employment information and opportunities provided by the university through various channels, such as labor market dynamics, job postings, and job search strategies. This dimension was clearly supported in the study and is consistent with existing research that highlights the critical role of informational support in job search success rates (Beehr et al., 2000; Jaidi et al., 2011). The Perceived Instrumental Support dimension encompasses practical job search tools and resources offered by the university, including resume writing guidance, interview skills training, and internship opportunities. Research indicates that instrumental support significantly enhances graduates’ employability and job search confidence (Martín-García et al., 2020). The Perceived Emotional Support dimension focuses on the psychological and emotional support provided by the university, such as career counseling, stress management, and psychological counseling services. These services play a crucial role in alleviating job search anxiety and enhancing job search motivation (Azmitia et al., 2013; Plessis et al., 2012). The Perceived Policy Support dimension includes policy-level measures provided by the university, such as employment guidance, entrepreneurial subsidies, and university-industry collaborations, which positively impact graduates’ employment quality and overall support evaluation (Cai, 2013).

The CFA results indicated that the 20 items accurately capture the unique characteristics of each dimension, and all fit indices for the four-factor model met the ideal standards, further confirming the structural validity of the PUESS. Specifically, the CFI and TLI indices ranged from 0.967 to 0.979, the RMSEA indices ranged from 0.046 to 0.053, and the SRMR indices ranged from 0.040 to 0.046, all meeting the excellent model fit criteria proposed by Cheung and Rensvold (1999).

Regarding reliability analysis, the Cronbach’s α coefficient of the PUESS was 0.94, and the test-retest reliability was 0.83, indicating high internal consistency and good temporal stability (Cronbach, 1951). Validity testing results comprehensively supported the structural and discriminant validity of the PUESS. The correlation coefficients between each factor and the total score were significantly higher than those among the factors themselves (Joseph et al., 2009). The Average Variance Extracted (AVE) and Composite Reliability (CR) for each factor met or exceeded psychometric standards. Additionally, the square roots of the AVE for all four factors were significantly greater than the maximum inter-factor correlations, further verifying the discriminant validity of the scale. This demonstrates that the PUESS can accurately and reliably measure graduates’ perceptions of university-based employment support and possesses high discriminant validity. Furthermore, the validity analysis provides strong evidence for the PUESS’s practical and theoretical utility. The scale demonstrated good criterion validity, as shown by its significant negative correlation with the Vocational Selection Anxiety Questionnaire for University Graduates. This indicates that higher perceptions of university employment support are a meaningful predictor of lower job-search anxiety, calibrating the scale against a critical outcome. Additionally, the PUESS showed good convergent validity through its significant positive correlation with the College Students’ Social Support Scale, confirming its alignment with the broader theoretical construct of social support. Crucially, this pattern of relationships underscores that the PUESS not only reflects general social support but also effectively captures graduates’ unique, employment-specific perceptions that are directly relevant to their transition outcomes, solidifying its utility as a targeted evaluation tool (Ye and Dai, 2008; Zhang and Chen, 2006; Zimet et al., 1988). The study also examined the measurement invariance of the PUESS across different gender, educational levels, and school types. The results indicated that the PUESS consistently measures the same constructs across diverse groups (Davidov et al., 2014), demonstrating good cross-group applicability (Cheung and Rensvold, 1999; Emerson et al., 2017). This finding provides a solid foundation for the future use of the PUESS among diverse graduate populations in various contexts.

In conclusion, the PUESS developed in this study exhibits excellent reliability, validity, and measurement invariance, making it a reliable tool for understanding and assessing university graduates’ perceptions of university-based employment support.

### 4.1 Implications

This study systematically revealed the multidimensional structure of university-based employment support by developing and validating the PUESS, offering a novel theoretical perspective for understanding the types of support perceived by graduates during their transition to the labor market and their subsequent impact. First, based on social support theory (Cohen and Wills, 1985; House et al., 1988), this study identified four core dimensions: Perceived Informational Support, Perceived Instrumental Support, Perceived Emotional Support, and Perceived Policy Support. This structure not only extends the application of social support theory to the fields of education and career development but also enriches the theoretical connotations of existing research on university-based employment support.

Moreover, the PUESS provides a precise and effective measurement tool for university administrators and career counselors, facilitating the comprehensive identification of graduates’ specific needs for employment support. This scale offers crucial empirical evidence for universities to formulate targeted career guidance strategies and optimize policies. The four core dimensions of the PUESS offer a systematic framework for understanding the synergistic effects of different types of support in enhancing graduates’ job readiness and market adaptability. The findings indicate that various types of support have differing levels of influence in improving students’ employability, alleviating job search stress, and promoting career development. This underscores the importance of comprehensive support strategies, suggesting that universities should provide diverse employment support services tailored to the individual needs of students. Specifically, universities can effectively enhance graduates’ career competitiveness and market adaptability by improving information dissemination channels, offering practical job search training, strengthening psychological and emotional support, and formulating flexible employment policies. Furthermore, the application of the PUESS can assist universities in allocating resources more rationally and effectively while providing graduates with more personalized and precise career guidance in practice, thereby increasing their success rates and satisfaction during the job search process. These practical implications offer feasible strategies for universities to optimize their employment support systems and enhance the employment quality of their graduates.

### 4.2 Limitations and future directions

Despite the significant achievements in developing and validating the PUESS, several limitations remain.

First, although the sample included university graduates from multiple provinces and various academic disciplines across China, the sample size was relatively small, which may have affected the statistical power and external validity of the findings. Future research should aim to expand the sample size and increase data representativeness to enhance the generalizability and robustness of the results.

Second, the study employed a cross-sectional design, which limits the ability to examine the dynamic, long-term effects of university-based employment support on graduates’ career development. Future studies could adopt a longitudinal design to track changes in graduates’ perceptions of employment support over different career stages and explore the long-term impact of such support on career success.

Third, the Phase 1 interview design primarily relied on student respondents and included some closed-ended questions. While this approach effectively captured students’ perceived experiences of university-based support, it may have constrained the depth of insights regarding the institutional and operational mechanisms of policy support. Consequently, the conceptualization of this dimension might not fully reflect the perspectives of policy designers (e.g., career center officers or student affairs administrators) and practitioners. Future research should therefore incorporate broader participant groups and employ more open-ended inquiry to enrich the understanding of policy support in higher education employment contexts.

Lastly, the study primarily relied on self-reported data, which may introduce issues such as social desirability bias and common method bias. To address this, future research could integrate data from multiple sources (e.g., evaluations from teachers and career counselors) and qualitative research methods to further validate the reliability and validity of the PUESS and gain a deeper understanding of graduates’ actual experiences with university-based employment support.

## 5 Conclusion

The findings of this study demonstrate that the PUESS is an effective and psychometrically robust assessment tool. The PUESS exhibits strong reliability and validity, and shows measurement invariance across gender, educational levels, and school types. It encompasses four dimensions–Perceived Informational Support, Perceived Instrumental Support, Perceived Emotional Support, and Perceived Policy Support–comprising a total of 20 items, which comprehensively capture various aspects of university-based employment support for graduates. The PUESS can be widely applied in related research, providing a scientific basis and reliable tool for the evaluation and optimization of university employment support mechanisms.

## Data Availability

The original contributions presented in this study are included in this article/supplementary material, further inquiries can be directed to the corresponding author.
